# Identifying Malaria Hot Spots

**DOI:** 10.1093/infdis/jix330

**Published:** 2017-07-17

**Authors:** Nicholas J White

**Affiliations:** Mahidol-Oxford Tropical Medicine Research Unit, Faculty of Tropical Medicine, Mahidol University, Bangkok, Thailand

**Keywords:** malaria, *Plasmodium falciparum*, epidemiology

**(See the major article by Mogeni et al, on pages 1091–8.)**

A century ago, malaria was prevalent across much of the inhabited world. With improvements in housing, environmental management, access to antimalarial drugs (quinine, followed by chloroquine), and use of the insecticide DDT, malaria was driven from Europe, North America, and the Union of Soviet Socialist Republics. Building on these early successes, the World Health Organization launched the first malaria global eradication campaign in 1955. The campaign failed to eradicate malaria in the tropics, and it was abandoned in 1969. Over the next 3 decades, the incidence of malaria increased again across much of the tropical world [1]. We are now trying again to eliminate malaria. Where effective control measures (artemisinin combination therapies, insecticide-treated nets, and, in some areas, insecticides) are being deployed, malaria incidences are decreasing. In contrast, in difficult-to-reach conflict zones, the supply of even the most basic diagnostic tools and effective antimalarial treatments remains sporadic, and malaria transmission presumably still remains high [2]. In some, more readily accessible parts of Africa, transmission has fallen markedly, and the epidemiology of malaria is beginning to resemble that described traditionally in the Americas and much of Asia [1]. Within these areas of generally low seasonal transmission sit small foci of more intense transmission that sustain malaria [3, 4]. These small foci, referred to “hot spots,” are major impediments to malaria elimination, so their identification is important as countries augment their malaria elimination activities. But how should we identify malaria hot spots in settings of low transmission?

Over 80 years ago, the Dutch malariologists showed that, in an area of *Plasmodium vivax* malaria endemicity in the Netherlands, most of those infected were asymptomatic [5]. They demonstrated that the majority of these “healthy carriers” had microscopy-assessed parasite densities between 1000 and 8000 parasites/mL of blood. No one today can match this degree of microscopy assiduity, but polymerase chain reaction (PCR) analysis provides a satisfactory alternative. PCR studies from several areas of low, unstable malaria transmission have revealed a much higher prevalence of asymptomatic infection than previously estimated from conventional diagnostic assays (ie, microscopy and *Pf*HRP2-based rapid tests). Findings of ultrasensitive quantitative PCR in recent studies from the Greater Mekong subregion suggest that the geometric mean parasite density of both *P. vivax* and *Plasmodium falciparum* in these asymptomatic infections approximates 5000 parasites/mL, consistent with observations from the early Dutch work and well below the conventional levels of microscopy detection [6]. In this issue of *The Journal of Infectious Diseases*, Mogeni et al report a detailed and comprehensive epidemiological evaluation of *P. falciparum* hot spots on the Kenyan coast, an area where malaria incidence has decreased markedly in recent years [7]. They show that whereas population screening with conventional diagnostic assays is probably sufficient for hot-spot identification in areas of higher transmission, the more sensitive PCR assay is needed to identify hot spots in areas of lower transmission. They conclude that malaria control programs should consider PCR testing for hot-spot identification when the prevalence of malaria parasite infection is low, as it will be en route to elimination [1, 7]. Potentially simpler *Pf*HRP2-based rapid tests with increased sensitivity are being developed, but, as Mogeni et al point out, whether they will be sufficiently sensitive to identify malaria hot spots still remains to be determined. Indeed, whether the current widely used filter paper–based PCR methods, using small blood sample volumes with limits of detection that are close to the geometric mean parasite density in asymptomatic individuals, are sufficiently sensitive to identify all hot spots is also uncertain. And what should be done when the hot spot is identified? The first step (if not already in place) should be provision of access to rapid diagnosis and effective treatment of symptomatic malaria. If it is assumed that everyone has access to an insecticide-treated bed net, should the next step be treatment of everyone living in the hot spot, treatment only of those identified as infected, or treatment of all those in infected households? And then what? There is as yet no consensus, and indeed there probably need to be different strategies for different epidemiological contexts [1]. Unfortunately, time may not be on our side, as worsening antimalarial drug resistance in Southeast Asia and increasing pyrethroid insecticide resistance in Africa threaten the ambitious malaria elimination targets set by many malaria-affected countries.

## Note

***Potential conflicts of interest.*** Author certifies no potential conflicts of interest. The author has submitted the ICMJE Form for Disclosure of Potential Conflicts of Interest. Conflicts that the editors consider relevant to the content of the manuscript have been disclosed.

## References

[1] Snow RW Global malaria eradication and the importance of *Plasmodium falciparum* epidemiology in Africa. BMC Med 2015; 13:23. 25644195 10.1186/s12916-014-0254-7 PMC4314741

[2] Bennett A, Bisanzio D, Yukich JO Population coverage of artemisinin-based combination treatment in children younger than 5 years with fever and *Plasmodium falciparum* infection in Africa, 2003–2015: a modelling study using data from national surveys. Lancet Glob Health 2017; 5:e418–27. 28288746 10.1016/S2214-109X(17)30076-1 PMC5450656

[3] Srivastava A, Nagpal BN, Joshi PL, Paliwal JC, Dash AP Identification of malaria hot spots for focused intervention in tribal state of India: a GIS based approach. Int J Health Geogr 2009; 8:30. 19457227 10.1186/1476-072X-8-30 PMC2688490

[4] Bousema T, Drakeley C, Gesase S Identification of hot spots of malaria transmission for targeted malaria control. J Infect Dis 2010; 201:1764–74. 20415536 10.1086/652456

[5] Swellengrebel NH, De Buck A. Malaria in the Netherlands. Amsterdam: Scheltema & Holkema, 1938.

[6] Imwong M, Stepniewska K, Tripura R Numerical distributions of parasite densities during asymptomatic malaria. J Infect Dis 2016; 213:1322–9. 26681777 10.1093/infdis/jiv596 PMC4799672

[7] Mogeni P, Williams TN, Omedo I Detecting malaria hotspots: a comparison between RDT, microscopy and polymerase chain reaction. J Infect Dis 2017. In this issue. 10.1093/infdis/jix321 PMC5853881 28973672

